# Oral nifedipine and phytosterol, intravenous nicardipine, and oral nifedipine only: Three-arm, retrospective, cohort study for management of severe preeclampsia

**DOI:** 10.1515/biol-2022-0581

**Published:** 2023-05-24

**Authors:** Shanduo Ma, Limei Zhu, Tiantian Zhou, Ting Qi, Weijun Wang

**Affiliations:** Department of Obstetrics and Gynecology, The First People’s Hospital of Lianyungang, No. 182 of Tongguan North Road, Haizhou District, Lianyungang, 222061 Jiangsu, China; Department of Clinical Laboratory, The First People’s Hospital of Lianyungang, Lianyungang, 222061 Jiangsu, China

**Keywords:** blood pressure, gestational age, nicardipine, nifedipine, phytosterol, preeclampsia, pregnancy

## Abstract

The treatment of preeclampsia is delivering women and extracting the placenta, but the Chinese Society of Obstetrics and Gynecology guidelines do not recommend the delivery of babies without severe features. The objectives of the study were to compare the effectiveness and safety of nifedipine and phytosterol in combination with nicardipine for the management of severe preeclampsia. Women (19–32 years; gestation age ≥ 30 weeks) with the complication of severe preeclampsia have received 10 mg of oral nifedipine (pregnant women received 10 mg of oralnifedipine, *n* = 112) or 1 mg/h intravenous nicardipine (pregnant women received 1 mg/h intravenous nicardipine (ND cohort), *n* = 115) or oral 10 mg nifedipine and 500 mg phytosterol (pregnant women received oral 10 mg nifedipine and 500 mg phytosterol (np cohort), *n* = 111) until 150/100 mmHg blood pressure was achieved. The time required to achieve the desired blood pressure control was 13 minutes shorter in the NP cohort compared to the NF (*p* < 0.0001, *t* = 11.605), and 3 minutes shorter compared to the ND (*p* < 0.0001, *t* = 2.79) cohorts. Stillbirths were reported in 14 (13%), 28 (24%), and 10 (9%) infants, and 13 (12%), 26 (23%), and 10 (9%) infants died from the NF, ND, and NP cohorts, respectively. The undesirable tocolytic effect was reported in 17 (15%) women of the ND cohort. Phytosterol with nifedipine has a synergistic or additive effect on the management of preeclampsia with fewer adverse outcomes.

## 1 Introduction

Preeclampsia is the leading cause of maternal morbidity and mortality [1] due to complications that have arisen from severe hypertension in pregnant women [2]. Therefore, in pregnant women with severe hypertension, blood pressure is required to be controlled [3]. Preeclampsia is reported in pregnancy after 20 weeks of gestation and is characterized by high blood pressure (systolic blood pressure >160 mmHg/diastolic blood pressure >110 mmHg) and proteinuria (≥300 mg/24 h) [2]. Preeclampsia leads to the development of eclampsia [4]. The prevalence of eclampsia in China is 0.2% [5].

The treatment of preeclampsia is delivering the patient and extracting the placenta [6] but when the blood pressure of a pregnant woman is higher (>160/110 mmHg), antihypertensive agents are administered to stabilize the patient [2]. However, these recommendations are not uniform with many recommended guidelines for a lower threshold (the National Institute for Health and Care Guidelines, World Health Organization [7] HYPERTENSION Canada, the International Society for the Study of Hypertension in Pregnancy, and the International Federation of Gynecology and Obstetrics guidelines [4]). Chinese guidelines of the Chinese Society of Obstetrics and Gynecology [8] have different diagnostic criteria for preeclampsia than the other international guidelines and are not recommending the delivery of babies in pregnant women with preeclampsia without severe features. Women should not give birth until 37 weeks gestation, which leads to delayed delivery in women with preeclampsia. In severe preeclampsia, termination of pregnancy is recommended at 34 weeks or even earlier depending on the maternal–fetal state. Also, there is a lack of a detailed management plan for preeclampsia in the guidelines of the Chinese Society of Obstetrics and Gynecology [8].

The American College of Obstetricians and Gynecologists 2013 [9] and the National Institute for Health and Care Guidelines [10], are recommending oral nifedipine (a calcium channel blocker), and intravenous labetalol (a combined *α*- and β-adrenergic blocker), but do not recommend intravenous nicardipine (a calcium channel blocker) in severe preeclampsia. Intravenous labetalol provides comparatively faster management of blood pressure and is successful in delaying further hypertensive crises after its administration in pregnant women with severe preeclampsia. However, it is expensive and has a higher chance of neonatal death [2,6,11]. While, oral nifedipine is comparatively less expensive and safe for neonates, and convenient for women [12]. It achieves slower management of high blood pressure [2,6,11]. Also, oral nifedipine is not successful in delaying further hypertensive crises after its administration in women with severe preeclampsia [13]. Therefore, the total dose for preeclampsia management is comparatively higher. The placebo-controlled, randomized trial reported the safety and superior efficacy of the combination of oral nifedipine and phytosterol (a natural compound) than nifedipine alone for the management of preeclampsia [14]. However, the mechanism of action responsible for the antihypertensive effect of phytosterol is unknown. For fast and adequate management of preeclampsia, intravenous nicardipine is used [15]. However, there are no adequate studies on intravenous nicardipine in pregnant females. It is used for the management of preeclampsia but animal studies have reported the secretion of nicardipine in breast milk [16]. Also, intravenous nicardipine interferes with the spontaneous induction of labor (undesirable tocolytic effect) before 34 weeks of gestation [17] and is only used when no other suitable option is available for preeclampsia and is not used in multiple pregnancies [18].

The objectives of the retrospective study were to compare the time and the amount of calcium channel blocker required to control the blood pressure to less than 150/100 mmHg, the time required for further hypertensive crisis after intervention(s), and the safety of the mother and fetus for oral nifedipine and phytosterol combination and oral nifedipine only with intravenous nicardipine in Chinese pregnant women with severe preeclampsia.

## 2 Materials and methods

### 2.1 Ethics approval and consent to participate

The designed protocol (TJMC220120 dated October 2, 2020) of the established study was approved by the First People’s Hospital of Lianyungang review board and the Chinese Society of Obstetrics and Gynecology. The study reporting adheres to the law of China and the V2008 the World Medical Association Declaration of Helsinki. Informed written consent was obtained from all subjects and/or their legal guardian(s) (routine protocol of the institute) before admission to hospitals regarding intervention(s), pathology, and participation in the study.


**Informed consent:** Informed consent has been obtained from all individuals included in this study.
**Ethical approval:** The research related to human use has been complied with all the relevant national regulations, institutional policies, and in accordance with the tenets of the Helsinki Declaration, and has been approved by the authors’ institutional review board or equivalent committee.

### 2.2 Inclusion criteria

Women (age >18 years) with the complication of severe preeclampsia diagnosed according to the report of the American College of Obstetricians and Gynecologists Task Force on Hypertension in Pregnancy [9] (gestation age >20 weeks, blood pressure >160/110 mmHg, and higher proteinuria ≥ 300 mg/24 h) and who had needed to receive treatment for the management of high blood pressure during their pregnancy were included in the analysis after getting written approval from the authorities.

### 2.3 Exclusion criteria

Women who had HELLP syndrome (Hemolysis, elevated liver enzymes, and low platelet count), diabetes, and a history of heart disease(s) were excluded from the analysis. Women who had a history of using other relevant drug therapies were excluded from the analysis.

### 2.4 Sample size calculation

The sample size (OpenEpi, Version 3, 01, Open-Source Epidemiologic Statistics for Public Health, USA) was calculated based on the assumption that 80 ± 13% of women achieved less than 150/100 mmHg blood pressure after intervention(s), 80% power (*β* = 0.2), and two-sided 5% type-I error (*α* = 0.05), and 95% confidence interval. This is a retrospective study of all eligible cases between January 2017 and December 2019. A “power analysis” i.e., what differences are achievable was preferred for sample size calculation.

### 2.5 Cohort

The oral nifedipine (NF) cohort consisted of 112 women who received immediate-release 10 mg tablet of oral nifedipine (Procardia, Pfizer, New York, NY, USA) at 15 min time intervals until 150/100 mmHg blood pressure was achieved or a maximum of up to five doses [2]. The nicardipine infusion (ND) cohort consisted of 115 women who received a continuous infusion of 1 mg/h of nicardipine (Cardene, PDL BioPharma, Inc., Redwood City, CA, USA) and the dose was increased by 0.5 mg at every hour (maximum 4 mg/h) until 150/100 mmHg blood pressure was achieved [16]. The oral nifedipine and phytosterol (NP) cohort consisted of 111 women who received immediate-release 10 mg of oral nifedipine tablet and oral 500 mg phytosterol powder (Shanghai Fudan Fuhua Pharmaceutical Co., Ltd, Shanghai, China; each 500 mg phytosterol powder contains 450 mg β-sitosterol) at 15 min time interval until 150/100 mmHg blood pressure was achieved or maximum up to five doses [14]. The different treatments for the management of severe preeclampsia were decided as per the preference of the concerned obstetrician(s). The very structured dosing schedule is an institutional protocol (not published yet). None of the women received magnesium sulfate. Once control was achieved, a maintenance dose (10 mg of oral nifedipine/8 h) was initiated in all women of all cohorts.

### 2.6 Demographical and clinical parameters

Data regarding socioeconomic status, height, weight, and blood pressure at the time of admission to the hospital were retrospectively collected from institutional records of women. Blood pressure was measured using the Sphygmomanometer (MDF^®^ Calibra^®^, MDF Instruments Direct, Inc., Rincon, PR, USA) during treatment and at the time of admission.

### 2.7 Outcome measures

Time and amount of the calcium channel blocker required to control blood pressure to less than 150/100 mmHg and time required for further hypertensive crisis (blood pressure >160/110 mmHg, and higher proteinuria ≥300 mg/24 h) after intervention(s) were retrospectively collected from the institutional records of women (the primary outcomes). All women have graphs that report differences in blood pressure per minute. Also, adverse effects and the infant dose of medication were retrospectively collected from the institutional records of women (the secondary outcomes). Proteinuria was measured in terms of mg/24 h from the urine sample.

### 2.8 Adverse effects

Treatment-emergent adverse effects for women and their infants were retrospectively collected from the institutional records of women and infants.

### 2.9 Relative infant dose of calcium channel blocker

Data on the concentration of calcium channel blockers in breast milk was collected from institutional records of women. The relative infant dose of calcium channel blocker was calculated as per equation (1). It was estimated that the maximum milk consumed by the infant was 150 ± 10 mL/day. Less than 10% of the relative infant dose of calcium channels was considered safe [16]. The milk samples were taken as soon as they started secreting from the breast of the women.

(1) \% {\rm{Relative\; infant\; dose\; of\; calcium\; channel\; blocker}}=\frac{{\rm{The\; concentration\; of\; calcium\; channel\; blocker\; in\; breast\; milk}}\left(\frac{{\rm{mg}}}{{\rm{mL}}}\right)\left\times {\rm{Volume\; of\; milk\; ingested}}\left({\rm{mL}})}{{\rm{Maternal\; dose\; of\; calcium\; channel\; blocker}}\left({\rm{mg}})}\times 100.]

### 2.10 Statistical analysis

InStat^®^ 3.01, GraphPad Software, San Diego, CA, USA was used for statistical analysis purposes. Categorical variables are presented as frequency (percentages) and continuous variables are presented as mean value ± standard deviation (SD). Histograms and plots of normality were used to check for a normal distribution of the data. One-way analysis of variance (ANOVA) was performed for continuous variables and the Chi-square test for independence (*χ* ^*2*^-test) was performed for categorical data. The Bonferroni multiple comparisons test was used for *post hoc* analysis (if the value of *t* is greater than 2.4, then the *p*-value is less than 0.050). Univariate followed by multivariate analyses were performed between the demographical and clinical conditions of women and treatment received by women for adverse infant outcomes [19]. All results were considered significant if the *p*-value was reported as less than 0.050.

## 3 Results

### 3.1 Study population

From 15 January 2017 to 19 December 2019, a total of 394 pregnant women (age >18 years) were diagnosed with the complication of severe preeclampsia and needed to receive treatment for the management of high blood pressure at the Department of Obstetrics and Gynecology of the First People’s Hospital of Lianyungang, Lianyungang, Jiangsu, China, and the referring hospital. Among 394 pregnant women, one woman had a history of heart failure, 13 women had HELLP syndrome, and 42 women had diabetes. Therefore, data of these women (*n* = 56) were excluded from the analysis.

Data of the demographical and clinical parameters of women at the time of admission, the time and amount of the calcium channel blocker required to control blood pressure to less than 150/100 mmHg, the time required for further hypertensive crisis after admiration of drug(s), and treatment-emergent adverse effects for women and their infants for a total of 338 women and their infants were collected from the institutional records of women and infants and were analyzed.

### 3.2 Demographical and clinical parameters

The age range of the included women was 19–32 years. All the included women had a gestation age of 30 weeks or more, a singleton pregnancy, higher blood pressure (>160/110 mmHg), and higher proteinuria (≥300 mg/24 h) at the time of hospital admission. A total of 19 (17%), 16 (14%), and 28 (25%) women in the pregnant women received 10 mg of oral nifedipine (NF cohort), pregnant women received 1 mg/h intravenous nicardipine (ND cohort), and pregnant women received oral 10 mg nifedipine and 500 mg phytosterol (NP cohort), respectively, had gestation age <32 weeks at the time of hospital admission. A total of 48 (43%), 49 (43%), and 55 (50%) women in the NF cohort, ND cohort, and NP cohort, respectively, had gestation age <34 weeks at the time of hospital admission. There were no significant differences in the demographical and clinical parameters of women (*p* > 0.050 for all, *χ* ^*2*^-test, or ANOVA) at the time of hospital admission. None of these women had aspirin prophylaxis before hospital admission. The details of the demographical and clinical parameters of women at the time of hospital admission are presented in Table 1.

**Table 1:** Demographical, clinical, and socioeconomic parameters of women at the time of admission to the hospital

Parameters		Cohorts					
		NF	ND	NP			
Number of women included in the analysis		112	115	111	Comparisons		
Treatment(s) for the management of high blood pressure		Oral nifedipine	Intravenous nicardipine	Oral nifedipine + oral phytosterol	*p*-value	*F*-value	Df
Maternal age (years)	Minimum	20	19	20	0.064 (ANOVA)	2.77	N/A
	Maximum	31	30	32			
	Mean value ± SD	25.71 ± 3.56	24.71 ± 3.44	25.65 ± 3.72			
Gestation age (weeks)	Minimum	30	30	30	0.429 (ANOVA)	0.85	N/A
	Maximum	38	39	38			
	Mean value ± SD	34.18 ± 2.51	34.31 ± 2.48	33.88 ± 2.62			
	<32 weeks	19 (17%)	16 (14%)	28 (25%)	0.079 (*χ* ^*2*^-test)	N/A	1
	<34 weeks	48 (43%)	49 (43%)	55 (50%)	0.496 (*χ* ^*2*^-test)	N/A	1
Systolic blood pressure (mmHg)		169.88 ± 4.74	170.50 ± 4.32	169.23 ± 4.74	0.118 (ANOVA)	2.15	N/A
Diastolic blood pressure (mmHg)		118.96 ± 3.72	119.44 ± 3.73	118.95 ± 3.67	0.524 (ANOVA)	0.65	N/A
Heart rate (beats/min)		81.79 ± 4.22	81.67 ± 4.40	81.42 ± 4.29	0.807 (ANOVA)	0.21	N/A
Body mass index (kg/m^2^)		25.12 ± 3.15	24.89 ± 3.67	25.41 ± 4.01	0.559 (ANOVA)	0.54	N/A
Proteinuria (mg/24 h)		310.48 ± 6.28	311.84 ± 4.53	311.97 ± 5.37	0.078 (ANOVA)	0.23	N/A
Socioeconomic status	Poor	31 (28%)	41 (36%)	42 (38%)	0.334 (*χ* ^*2*^-test)	N/A	4
	Middle	49 (44%)	39 (34%)	35 (32%)			
	High	32 (28%)	35 (30%)	34 (30%)			

Categorical variables are presented as frequency (percentages) and continuous variables are presented as mean value ± SD.

One-way ANOVA was performed for continuous variables and the Chi-square test for independence was performed for categorical data.

*p*-value < 0.05 was considered as significant.

*χ* ^*2*^-test: The Chi-square test for independence, N/A: Not applicable, Df: Degree of freedom.

### 3.3 Outcome measures

#### 3.3.1 Time required to control blood pressure to less than 150/100 mmHg

**Figure 1:**
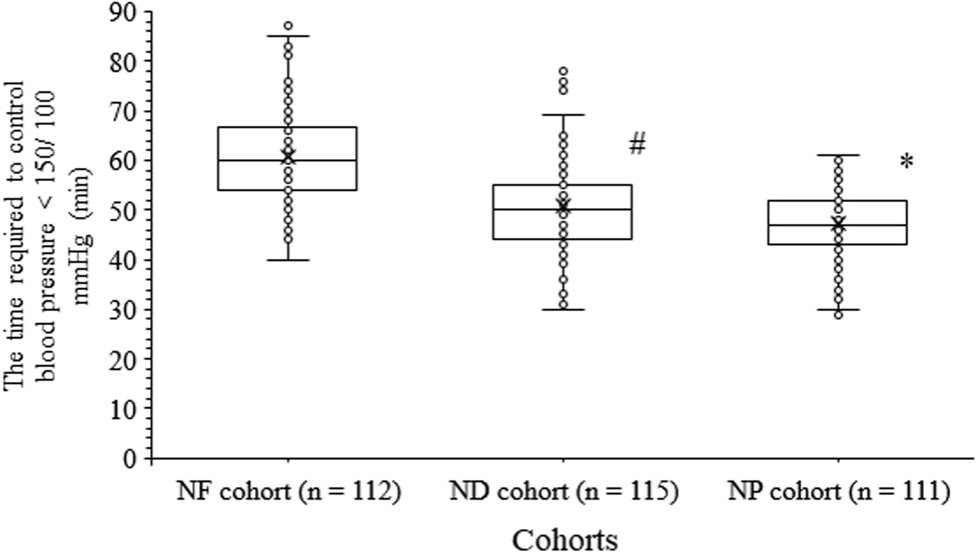
The time required to control blood pressure to less than 150/100 mmHg. * indicates shorter values than those of the NF and the ND cohorts. ^#^ indicates shorter values than those of the NF cohort. Box represents mean value.

All women had responded to the treatment. The time required to control blood pressure to less than 150/100 mmHg for women of the NP cohort was shorter than those of the NF (47.42 ± 6.99 min/woman vs 60.83 ± 9.07 min/woman, *p* < 0.0001, *t* = 11.6, ANOVA/Bonferroni test) and the ND (47.42 ± 6.99 min/woman vs 50.63 ± 9.56 min/woman, *p* < 0.0001, *t* = 2.8, ANOVA/Bonferroni test) cohorts. Also, the time required to control blood pressure to less than 150/100 mmHg for women of the ND cohort was shorter than those of the NF cohort (*p* < 0.0001, *t* = 8.9, ANOVA/Bonferroni test; Figure 1).

#### 3.3.2 Amount of calcium channel blocker required to control blood pressure to less than 150/100 mmHg

**Figure 2:**
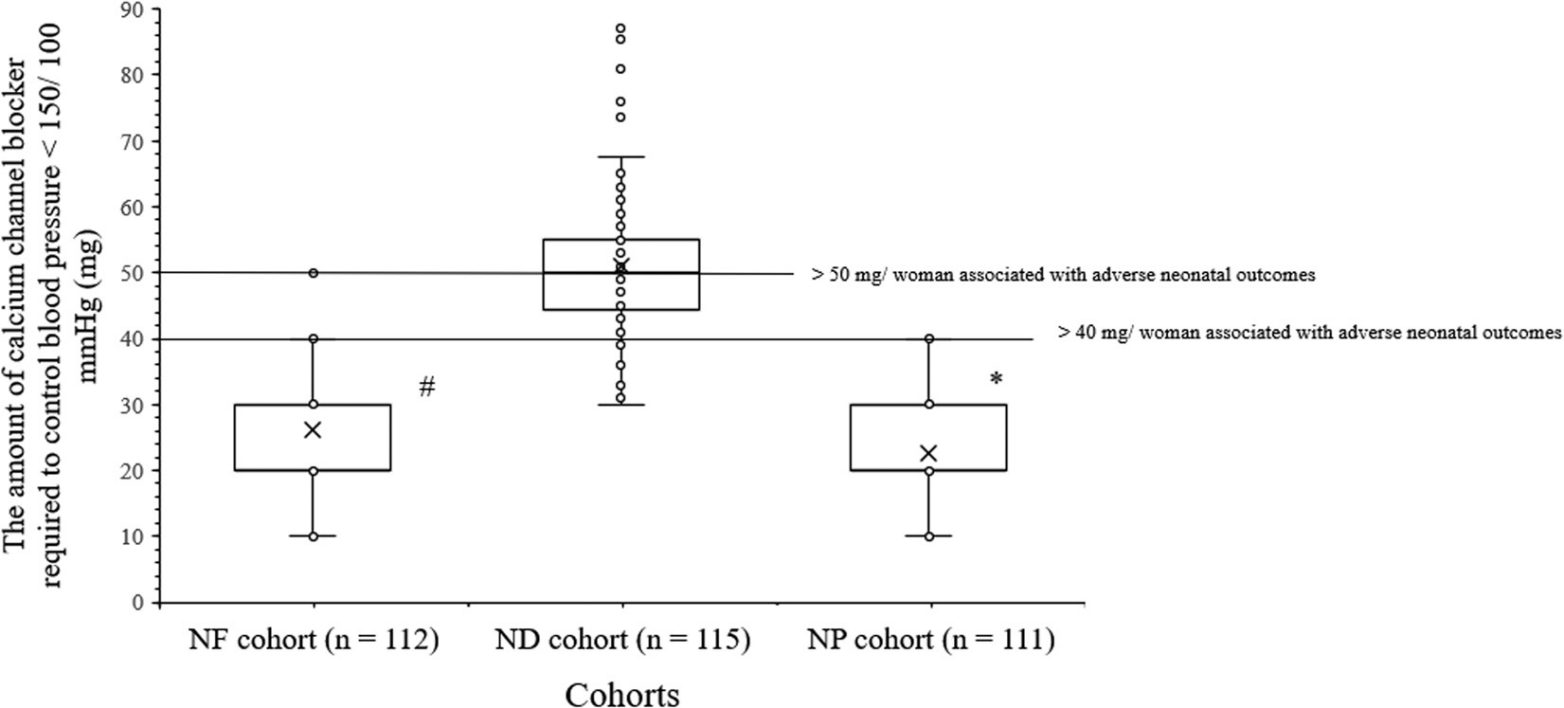
The amount of calcium channel blocker required to control blood pressure to less than 150/100 mmHg. * indicates shorter values than those of the NF and the ND cohorts. ^#^ indicates shorter values than those of the ND cohort. Box represents mean value.

The amount of calcium channel blocker required to control blood pressure to less than 150/100 mmHg was fewer for women of the NP cohort than those of the NF (22.52 ± 8.36 mg/woman vs 26.16 ± 10.68 mg/woman, *p* < 0.0001, *t* = 2.7, ANOVA/Bonferroni test) and the ND (22.52 ± 8.36 mg/woman vs 51.05 ± 10.75 mg/woman, *p* < 0.0001, *t* = 21.4, ANOVA/Bonferroni test) cohorts. The amount of calcium channel blocker required to control blood pressure to less than 150/100 mmHg was fewer for women of the NF cohort than those of the ND cohort (*p* < 0.0001, *t* = 18.7, ANOVA/Bonferroni test, Figure 2).

#### 3.3.3 Time required for further hypertensive crisis

**Figure 3:**
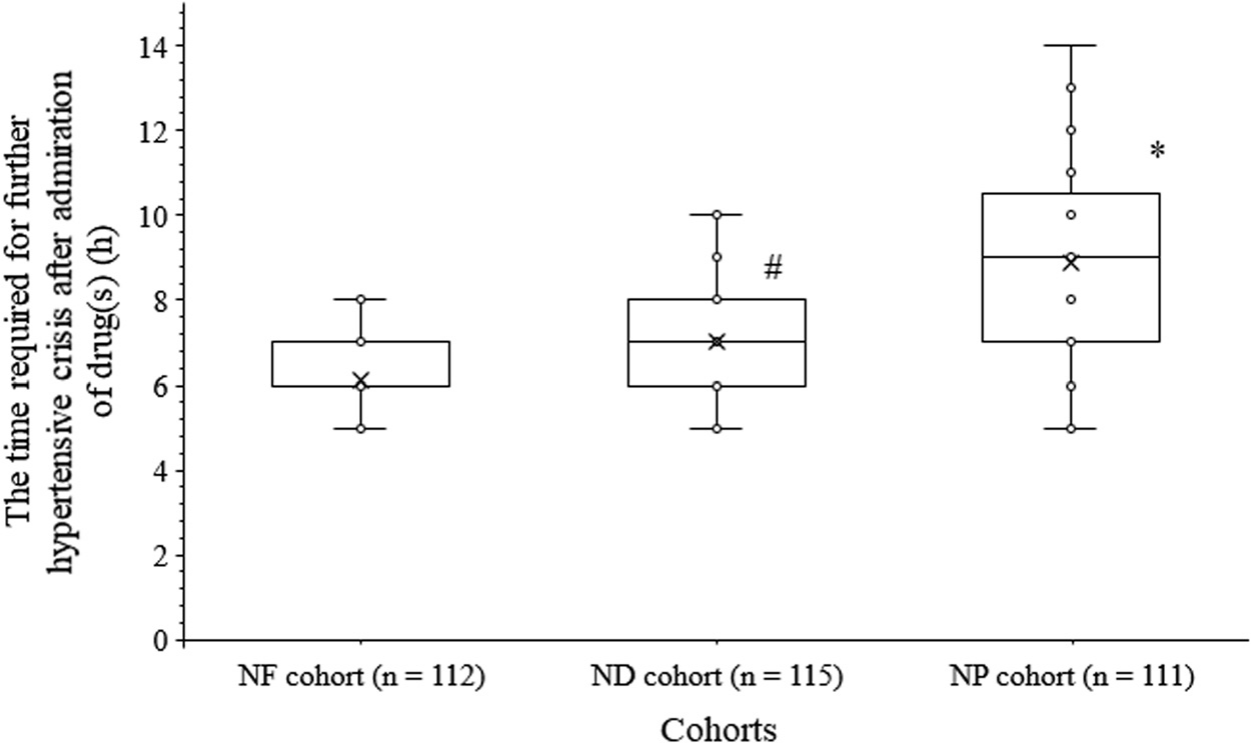
The time required for further hypertensive crisis after administration of the drug(s). * indicates longer values than those of the NF and the ND cohorts. ^#^ indicates longer values than those of the NF cohort. Box represents mean value.

The time required for further hypertensive crisis (the time until the next hypertensive crisis) in women after administration of the drug(s) was longer for the NP cohort than those of the NF (8.90 ± 2.07 h/woman vs 6.13 ± 0.87 h/woman, *p* < 0.0001, *t* = 13.8, ANOVA/Bonferroni test) and ND (8.90 ± 2.07 h/woman vs 7.03 ± 1.30 h/woman, *p* < 0.0001, *t* = 4.6, ANOVA/Bonferroni test) cohorts. Also, the time required for the further hypertensive crisis in women after administration of drug(s) was longer for the ND cohort than those of the NF cohort (*p* < 0.0001, *q* = 9.4, ANOVA/Bonferroni test, Figure 3).

### 3.4 Adverse effects

There were no significant differences in results of maternal vomiting, tachycardia, headache, dizziness, shortness of breath, and frequencies of intensive care unit admission for the infants among cohorts (*p* > 0.050 for all, ANOVA/Bonferroni test or *χ* ^*2*^-test). The undesirable tocolytic effect was reported in 2 (2%), 17 (15%), and 1 (1%) women of the NF, ND, and NP cohorts, respectively. Induction of labor was attempted in all women who had faced the tocolytic effect. Stillbirths were reported in 14 (13%), 28 (24%), and 10 (9%) infants of the NF, ND, and NP cohorts, respectively. Death was reported in 13 (12%), 26 (23%), and 10 (9%) infants of the NF, ND, and NP cohorts, respectively (Table 2).

**Table 2:** Short-term effects of treatments on maternal and infants’ outcomes

Effects		Cohorts			Comparison					
		NF	ND	NP						
Numbers of women included in the analysis		112	115	111	*p*-value	*F*-value	Df	*t*-value		
Treatment(s) for the management of high blood pressure		Oral nifedipine	Intravenous nicardipine	Oral nifedipine + oral phytosterol				NF vs ND	NF vs NP	ND vs NP
Maternal nausea		8 (7%)	6 (5%)	5 (5%)	0.677 (ANOVA)	0.39	N/A	N/C	N/C	N/C
Maternal vomiting		4 (4%)	4 (3%)	2 (2%)	0.715 (ANOVA)	0.34	N/A	N/C	N/C	N/C
Maternal tachycardia		2 (2%)	7 (6%)	2 (2%)	0.109 (ANOVA)	2.23	N/A	N/C	N/C	N/C
Maternal headache		6 (5%)	4 (4%)	2 (2%)	0.359 (ANOVA)	1.03	N/A	N/C	N/C	N/C
Maternal dizziness		4 (4%)	5(4%)	2 (2%)	0.547 (ANOVA)	0.60	N/A	N/C	N/C	N/C
Maternal shortness of breath		1 (1%)	1 (1%)	1 (1%)	0.999 (ANOVA)	0.01	N/A	N/C	N/C	N/C
Undesirable tocolytic effect		2 (2%)	17 (15%)^*^	1 (1%)	<0.0001 (ANOVA)	13.19	N/A	4.3	0.3	4.6
Infants’ birth weight (kg)		3.08 ± 0.41	2.99 ± 0.42	3.08 ± 0.11	0.078 (ANOVA)	1.07	N/A	N/A	N/A	N/A
Apgar score	4–6	25 (22%)	20 (17%)	20 (18%)	0.593 (*χ* ^*2*^-test)	N/A	2	N/C	N/C	N/C
	>6	87 (78%)	95 (82%)	91 (82%)						
NICU admission		34 (30%)	27 (23%)	22 (20%)	0.097 (ANOVA)	2.35	N/A	N/C	N/C	N/C
Stillbirths		14 (13%)	28 (24%)^*^	10 (9%)	0.003 (ANOVA)	5.78	N/A	2.5	0.7	3.2
Infants’ death		13 (12%)	26 (23%)^*^	10 (9%)	0.008 (ANOVA)	4.87	N/A	2.4	0.6	2.9

NICU: Neonatal intensive care unit.

Categorical variables are presented as frequency (percentages) and continuous variables are presented as mean value ± SD.

One-way ANOVA or *χ* ^*2*^-test was performed for statistical analysis.

The Bonferroni test was used for *post hoc* analysis.

*p*-value < 0.05 and *t* > 2.406 were considered as significant.

^*^Intravenous nicardipine-emergent adverse effects.

The effect was considered as “1” and the absence of effect was considered as “0” for statistical analysis.

*χ* ^*2*^-test: The Chi-square test for independence, N/A: not applicable, Df: degree of freedom, N/C: not calculated.

### 3.5 Risk of adverse infant outcomes

A dosage amount of total of 50 mg/woman or more of intravenous nicardipine required to control the blood pressure to less than 150/100 mmHg (*p* = 0.045, multivariate analysis) and 40 mg/woman or more of oral nifedipine required to control the blood pressure to less than 150/100 mmHg (*p* = 0.048, multivariate analysis) in women with less than 32 weeks of gestation age (*p* = 0.021, multivariate analysis) was associated with adverse infants’ outcomes. While this dosage amount in women with less than 34 weeks of gestation age was not associated with adverse infant outcomes (*p* = 0.502, multivariate analysis).

### 3.6 Relative infant dose of calcium channel blocker

The relative infant dose of calcium channel blocker in infants born to women in the NF cohort was 5.95 ± 1.74%, in the ND cohort was 10.07 ± 2.04%, and in the NP cohort was 4.64 ± 2.25%.

## 4 Discussion

This is a retrospective study comparing three cohorts of preeclamptic mothers treated with nifedipine alone, nifedipine plus phytosterol, and nicardipine infusion. Phytosterol was found to have a synergistic effect on the action of nifedipine in the time and amount of calcium channel blocker required to control blood pressure to less than 150/100 mmHg, the time until a further hypertensive crisis, and maternal and infants’ treatment-emergent adverse effects compared to oral nifedipine alone or intravenous nicardipine. The results of the preeclampsia management of the current study agreed with those of randomized clinical trials [13,14]. Oral nifedipine and intravenous nicardipine are common drugs used for the management of preeclampsia [3]. Phytosterol has a plant origin and it is proven to be safe during pregnancy [20]. Also, the additive or synergistic effect can delay further hypertensive crisis after the control of hypertension [21]. Phytosterol has been evaluated in a meta-analysis and was shown to reduce both systolic and diastolic blood pressure by an average of 1.55 and 0.84 mmHg, respectively, in data pooled from 19 randomized clinical trials [21]. It has been supported as a food supplement to reduce cardiovascular risks by organizations such as the United States Food and Drug Administration. This is still insufficient to recommend it to pregnant women since its safety profile has not been thoroughly investigated. Oral phytosterol with nifedipine provides a synergistic effect to control hypertension for the management of severe preeclampsia and advantages of a delay crisis in preeclampsia women.

The time required to control blood pressure to less than 150/100 mmHg for women suffering from preeclampsia was shorter and the time until a further hypertensive crisis occurred was longer in the case of intravenous nicardipine than oral nifedipine. Nifedipine is a very short-acting drug so the efficiency of lowering the blood pressure within a few minutes of administration relative to nicardipine is not very important, but once the blood pressure is down, the drug is so short-acting that keeping the blood pressure down will require other medications with a longer duration of action.

The amount of calcium channel blocker required to control blood pressure to less than 150/100 mmHg was less in the case of oral nifedipine than intravenous nicardipine. Moreover, controlled intravenous nicardipine was associated with undesirable tocolytic effects, stillbirths, and infant death. Women with severe preeclampsia have a higher risk of adverse pregnancy outcomes than women with chronic/gestational hypertension [22]. Severe intrauterine growth restriction and placental abruption are responsible for stillbirths and infant death [6]. The antihypertensive agent can reduce uteroplacental blood flow in women with less than 32 weeks of gestation age, which can in turn result in severe intrauterine growth restriction and placental abruption [23]. The reason for different outcome results for oral nifedipine and intravenous nicardipine is that different mechanisms of action for reduction in blood pressure i.e., oral nifedipine gradually decreases blood pressure while intravenous nicardipine reduces blood pressure by rapid onset of action [16]. Neonatal outcomes are better with oral nifedipine in the management of preeclampsia [22]. It is well known that a fast reduction in increased blood pressure may be associated with cardiotocographic signs of fetal distress. Intravenous nicardipine is superiorly effective compared to oral nifedipine in the management of preeclampsia but this treatment has associated risk for infants’ health, especially in women with less than 32 weeks of gestation age, and undesirable tocolytic effect.

The study reported that oral nifedipine, oral phytosterol, and intravenous nicardipine did not significantly affect maternal health. These results indicated that oral nifedipine, intravenous nicardipine, and oral phytosterol are safe for women suffering from severe preeclampsia. In the context of perinatal mortality, the other infants’ outcomes are also required to be considered to reach this conclusion.

The time required to control blood pressure for NP was the shortest among the three cohorts. The use of oral medications in severe preeclampsia is potentially problematic as some women may have altered sensorium or nausea/vomiting and be unable to safely swallow pills; also, oral medications such as short-acting nifedipine cause unpredictable reductions in blood pressure which at times can be very precipitous. Oral nifedipine plus phytosterol gradually and precipitously lower blood pressure than nicardipine and other medications that are recommended for use in severe preeclampsia such as hydralazine and labetalol. Further research is required to find out the paradoxical results of the current study with expected outcomes.

Relative infant dose of calcium channel blockers of infants of women who received intravenous nicardipine was reported higher than 10%. A total of 10% or more of the relative infant dose of any drug is not recommended because of the lower drug clearance capacity of infants [16]. The intravenous administration of nicardipine is the likely explanation for the higher relative infant dose. Nicardipine clearance in the placenta cannot be ruled out.

The absolute incidence of 9–24% stillbirth is very high. The ND cohort presented a very-high frequency of stillbirths and neonatal deaths (summing both, almost half of the women who received this medication lost their babies). The neonatal death rate was also so high among all women. This was not near zero. Although fetal monitoring is a normal policy in China, the setting has no fetal monitoring (this is a resource issue locally). This is an unacceptable fetal and infant death rate. It raises questions on possible unsafe management of pregnant women with preeclampsia especially in the average gestational age of 33–34 weeks. Intravenous nicardipine should not be a standard choice for the management of severe preeclampsia in pregnant women. Also, clear Chinese guidelines are required from the Chinese Society of Obstetrics and Gynecology for severe preeclampsia management.

None of the women received magnesium sulfate. Administration of magnesium sulfate is a clear recommendation, especially for severe preeclampsia before 32 weeks of gestation (the current study had 63 pregnant women with gestation less than 32 weeks) because its neuroprotective role for the newborn and anticonvulsant for pregnant women have been scientifically proven. This is a wrong approach, both ethically and methodologically. The worse perceptions and experiences of healthcare providers related to the administration of magnesium sulfate to the parent and the referring institutes restrict the use of magnesium sulfate for severe preeclampsia. Clear Chinese guidelines are required from the Chinese Society of Obstetrics and Gynecology for the use of magnesium sulfate for severe preeclampsia.

The starting dose of nicardipine used in the available research [16] cited by the current study was 0.5 mg/h, not 1 mg/h as described in the methods section of the current study. However, the time required to achieve blood pressure less than 150/100 mmHg for women of the ND cohort was 50.63 ± 9.56 min/woman. Previous studies demonstrated that nicardipine has a fast onset of action (within 10 min) and a short elimination half-life (2–5 min), resulting in a fast and controllable antihypertensive effect. Sufficient reduction in blood pressure occurs most of the time within 20 min if the initial infusion rate is set at 2–3 mg/h of nicardipine with increments of 0.5–1 mg/h. A relatively low initial dose (continuous infusion of 1 mg/h) may be the potential reason for the same. However, the dosage of nicardipine used (1 mg/h) in the current study differed from the majority of previous studies for the fast action of nicardipine due to severe preeclampsia.

The use of phytosterols with calcium channel blockers is an interesting idea and is worthy of investigation. However, the study was a retrospective study. The other limitations of the study, for example, the underlying mechanism for the synergistic effect of oral phytosterol with nifedipine is still not clear. It is hypothesized that oral phytosterol can decrease the production of matrix metalloproteinase-3 and matrix metalloproteinase-13 [24], which are responsible for preeclampsia [25]. A placebo-controlled trial is required to elucidate the mechanism of action responsible for the synergistic effect of oral phytosterol with nifedipine to control hypertension for the management of preeclampsia. The long-term effects of treatments on maternal and infant outcomes were not evaluated. The perinatal mortality rate for a cohort with a mean gestational age of 34 weeks (3.0 kg) seems high. The conditions of fetal surveillance, the utilization of c-sections, and the infants’ nursery capacity for respiratory support were not discussed. With data on phytosterol alone, it is inaccurate to state that the effects of nifedipine and phytosterol are truly synergistic as suggested or merely additive. Neither the dose of nifedipine or nicardipine was increased nor other drugs were added to avoid hypertensive crises. The possible justification is that the dose of the drug and intervention(s) were kept less to avoid any fetal effect. Importantly, in 2020, The American College of Obstetrics and Gynecology [26] published evidence-based clinical guidelines for the diagnosis and management of gestational hypertension and preeclampsia and recommended prophylactic aspirin between 12 and 28 weeks of gestation age. However, the current study data were from before the publication of these guidelines.

## 5 Conclusion

Intravenous nicardipine is superiorly effective to oral nifedipine for the management of preeclampsia but is associated with stillbirth, infant death, especially with less than 32 weeks of gestation age, and undesirable tocolytic effect. Oral nifedipine is generally used by physicians for the management of high blood pressure in severe preeclampsia but it has slower management of high blood pressure and risk of further hypertensive crisis after the control of hypertension. Also, oral nifedipine has the risk of adverse infant outcomes if 40 mg or more amount is used to control blood pressure to less than 150/100 mmHg. The current study provides evidence that oral phytosterol with nifedipine combination has a synergistic or additive effect to control hypertension for the management of preeclampsia with fewer risks of maternal and infant adverse outcomes, especially with less than 32 weeks of gestation age. Also, oral phytosterol appears safe for women and infants. The current study recommended clear Chinese guidelines to the Chinese Society of Obstetrics and Gynecology for severe preeclampsia management. The study is recommending a diet other than the mother’s milk to the infants if delivery is done under intravenous nicardipine to rule out nicardipine clearance in the placenta. There is already evidence of the effects of various antihypertensive medications on preeclampsia. Most of the available evidence is limited by the lack of controlled randomized trials, but over recent years some large series have been published that illustrate the advantages and disadvantages of the main available drugs concerning the main phenotypes of preeclampsia (i.e., early vs late forms). The current study contains information of interest but the retrospective design and the relatively small numbers limit the interpretations of the results. However, it adds substantially novel information to what is already known on the subject.
